# [^68^Ga]/[^188^Re] Complexed [CDTMP] Trans-1,2-Cyclohexyldinitrilotetraphosphonic Acid As a Theranostic Agent for Skeletal Metastases

**DOI:** 10.3389/fmed.2017.00072

**Published:** 2017-06-09

**Authors:** Ambika P. Jaswal, Virendra K. Meena, Surbhi Prakash, Ankita Pandey, Baljinder Singh, Anil K. Mishra, Puja P. Hazari

**Affiliations:** ^1^Division of Cyclotron and Radiopharmaceutical Sciences, Institute of Nuclear Medicine and Allied Sciences, Delhi, India; ^2^Nuclear Medicine and PET Centre, PGIMER, Chandigarh, India

**Keywords:** CDTMP, radionuclide, radiopharmaceutical, positron emission tomography imaging, ^68^Ga, ^188^Re

## Abstract

**Objective:**

Metastasis of the osseous tissue is one of the frequent and severe aggravations as a result of several neoplastic conditions, such as metabolic disorders, infections, and cancer. The objective of this study was to evaluate the pertinence of [^68^Ga]-trans-1,2-cyclohexyldinitrilo tetramethylene phosphonic acid (CDTMP) as a potential bone imaging agent for positron emission tomography (PET) applications as well as to assess [^188^Re]-CDTMP for bone pain palliation in metastatic skeletal disorders.

**Methods:**

^68^Ga complex of CDTMP was prepared at 80°C at pH 3.5, and ^188^Re complex of CDTMP was prepared at room temperature. [^68^Ga]-CDTMP complex was investigated as PET tracer while the therapeutic efficacy was assessed for [^188^Re]-CDTMP. Labeling efficiency, biodistribution, myelotoxicity, and imaging studies were carried out for the complexes synthesized. Both PET and MicroPET imaging studies were performed for [^68^Ga]-CDTMP whereas SPECT acquisitions were acquired for [^188^Re]-CDTMP. Data were analyzed semiquantitatively for all the scintigraphic scans obtained.

**Results:**

The radiolabeling efficiency was observed to be >70% for [^68^Ga]-CDTMP. High bone uptake of [^68^Ga]-CDTMP as compared to contralateral tissue was found in PET imaging in Balb/C mice and New Zealand rabbit; the similar result for bone uptake was correlated in the biodistribution study of the compound in BALB/c mice at different time intervals. Biodistribution experiments carried out in mice showed maximum uptake of 6.12 ± 1.22%ID/g at 45 min postinjection. For [^188^Re]-CDTMP, total skeletal uptake was 8.12 ± 1.11%ID/g observed at 1 h postinjection from biodistribution data. High renal uptake confirms renal route of excretion. A good hydroxyapatite binding too was seen for both the complexes. No evidence of destruction or adverse functioning of vital organs was observed for the ^188^Re complex.

**Conclusion:**

[^68^Ga]-CDTMP complex can be used as a promising PET bone imaging agent and [^188^Re]-CDTMP as a surrogate moiety for therapeutic application. Owing to the short half-life of ^68^Ga (68 min), cyclotron-independent radiopharmacy, fast clearance, and rapid renal excretion as evidenced in preclinical animal models. Very low myelotoxicity and highly selective bone uptake prove the potential of [^188^Re]-CDTMP for therapeutic application.

## Introduction

Abnormal growth associated with neoplastic conditions, such as anomalous metabolic disorders, contagions, and malignancies result in metastasis in the skeletal tissue. Bone metastasis is prevalent among the majority of patients with metastatic conditions, which results in a high percentage of mortality and morbidity. These skeletal-related events that involve hypercalcemia caused by malignancy, pathological fractures, etc., and thus arises the requirement for surgical intervention or radiation treatment in bone. Published reports indicate toward the early occurrence of bone metastases; a tumor disease, however, the condition remains clinically asymptomatic and is recognized rather late (1, 2). Bone imaging is one of the medical imaging techniques in nuclear medicine in which whole body metastasis assessment is performed using radiotracers such as [^99m^Tc]-phosphonates and [^18^F]-fluoride. In the current scenario, a variety of [^99m^Tc] based tracers are being used for skeletal metastasis evaluation. Radiolabeled phosphonates are of utmost interest in nuclear medicine for metastasis evaluation as well as for therapy as many common tumors are associated with metastasis. Bone-targeting radiopharmaceuticals labeled with β emitters are acceptable adjuvant to external beam therapy for treatment of bone pain in osteoblastic bone metastatic condition. It has been reported that bone pain from metastatic disease occurs more profoundly in patients with malignancies in organs such as prostate, breast, and lung. Bone imaging and bone pain palliation are two distinct procedures followed in nuclear medicine. Phosphonates and phosphonic acids show high affinity for inorganic hydroxide and oxide materials and hence explored as the ideal targeting groups for hydroxyapatite (HAp) and bone. Bisphosphonate moieties radiolabeled with therapeutic isotopes are the category of pharmaceuticals that are widely accepted options for treatment of osteoporosis; a condition associated with an imbalance between osteoblastic and osteoclastic activities. Minimum exposure and maximum resolution, which signify a minimum radiation burden to the patient, as well as minimization of interfering signals in the scintigraphic image, require high specific skeletal uptake of the radiotracer (3). Ga-68-based radiopharmaceuticals for imaging bone thus found interest over ^18^F-NaF because of its longer half-life of 110-min and high radiation burden imposed to the patient (4–6). A therapeutic agent employed in radionuclide therapy consists of two components; α particle, β particle or Auger electron-emitting radioisotope and the molecular targeting vector that ensures maximum accumulation at or in desired tumor cells (7).

Polyphosphonates (PPs) such as MDP (methylenediphosphonate), DPD (3,3-diphosphono-1,2-propandicarboxylic acid), and EDTMP (ethylenediamino-*N*,*N*,*N*′,*N*′-tetrakismethylenephosphoric acid) are already established as suitable ligands for targeting therapeutic radionuclide into bone tissue (8). Various radiopharmaceuticals are in clinical use like [^153^Sm]ethylene diamine tetramethylene phosphonate (Quadramet) and rhenium-186-hydroxyethylidenediphosphonic acid ([^186^Re]-HEDP), all having unique characteristics (9, 10).

As compared to [^99m^Tc]-PPs, [^18^F]-fluoride has potential advantages of high sensitivity and increased spatial resolution due to advanced positron emission tomography (PET) technology (11, 12), thus arises a need for ligand-based tracers with improved diagnostic accuracy for PET application. Apart from ^18^F, ^68^Ga is another potential radionuclide for PET imaging. Because of advances and commercial availability of ^68^Ge/^68^Ga generators, ^68^Ga-radiolabeled PET tracers have found considerable interest. [^68^Ga]-phosphonates for PET bone imaging earns special mention, because of its easy availability from an in-house generator thus, overcoming the requirement of an on-site cyclotron facility. [^68^Ga]-labeled phosphonates also prevail over the practical question of higher costs associated with the cyclotron-based production of [^18^F]-fluoride as compared to a generator-based production and easy radiopharmacy, along with the limitation of on-site cyclotron access.

^188^Re is known to possess an excellent therapeutic potential with a short half-life value of 16.9 h, maximum particle energy of 2.1 MeV, and 10 mm of maximum beta range in tissue (13). Availability of (^188^W/^188^Re) generator offers momentous advantages regarding easy access and convenience when compared to other therapeutic radionuclides in clinical use. We were thus motivated to evaluate already existed PP, CDTMP as an alternative for [^68^Ga]-labeled known compounds with expected good uptake in bone as well as a therapeutic alternative for bone pain palliation in skeletal metastasis. The present study focuses on *in vivo* evaluation of [^68^Ga]-CDTMP, *ex vivo* evaluation regarding biodistribution, blood kinetics of [^68^Ga]-CDTMP, and evaluation of [^188^Re]-CDTMP as a potential agent for therapy.

Development of [^68^Ga]-based phosphonate derivative for bone PET may provide a new insight both in imaging and diagnosis, as well as an alternative to molybdenum shortage along with overcoming the requirement of an on-site cyclotron facility.

## Materials and Methods

### Chemicals

Trans-1,2-cyclohexyldinitrilotetraaceticacid, monohydrate salt [cyclohexylenedinitrilotetraacetic acid (CDTA)], phosphorous acid, phosphorous trichloride, Na-HEPES buffer [4-(2-hydroxyethyl)-1-piperazineethanesulfonic acid], stannous chloride, methanol, and ethanol (HPLC grade) normal saline were purchased from Sigma-Aldrich Co. ^68^Ga used was eluted from in-house Eckert-Ziegler IGG100 (50 mCi) TiO_2_-based [^68^Ge] breakthrough <0.001%. ^188^Re was procured in readily usable form from Amersham Biosciences.

### Instrumentation

Radio-TLC was analyzed on Peak Simple 3.29 wavelength detector K-2001, Knauer, Germany, using isocratic pump SykamS Gallium-68 complex, HPLC was performed on semiautomated GE FXFN TracerLab integrated with radioactivity detection and UV detection at 240 nm [fixed −1,021, elution with methanol and water (25/80, v/v) mobile phase and flow rate of 2 mL/min on C-18 reversed phase Nucleosil column (250 mm × 10 mm, 5 μm)]. Mass spectra (ESI-MS in positive and negative ion mode) were performed on in-house Agilent 6310 system ion trap. Mass spectra (ESI-MS in positive and negative ion mode) were performed on in-house Agilent 6310 system ion trap.

### Animal Studies

All the animal studies were done according to approved protocols of Institutional animal ethics committee (CPCSEA Regn No. 8/GO/RBi/S/99). New Zealand white Rabbits (2–3 kg), Wistar rats (200 ± 10 g), and Balb/C mice (25 ± 3 g) were used in experiments. Animals were housed under a controlled temperature condition of 22 ± 2°C and normal diet *ad libitum*.

### PET Imaging Experiments

Both PET and MicroPET acquisitions were performed. PET Imaging data were acquired on GE Discovery STE 16 system (PET/CT) system using New Zealand rabbits. PET data were reconstructed using vendor provided ordered-subsets expectation maximization. MicroPET imaging was performed on GE FLEX Triumph MicroPET/SPECT/CT scanner, which consists of a MicroPET module (LabPET 4) with 2 m × 2 m × 10 m, LYSO/LGSO scintillators in an 8-pixel, quad-APD detector module arrangement. The rabbit was injected intravenously through ear vein whereas mice were injected through the tail vein, and imaging was done 45 min postinjection for [^68^Ga]-CDTMP. The subject was anesthetized with ketamine (4.8 mg/kg), and midazolam (1.2 mg/kg) administered intramuscularly and was placed prone on PET scanner (37°C). Image reconstruction was done using Amira 4.1.1. Semiquantitative analysis was performed using Amide Software.

### SPECT Imaging Experiment

[^188^Re]-CDTMP planar images were acquired on GE Infinia Hawkeye System with NaI(Tl) crystal, Medium Energy General Purpose Collimator, 7.4 mm resolution at 10 cm and planar pixel size of 4.4 mm.

### Synthesis

**Figure 1:**
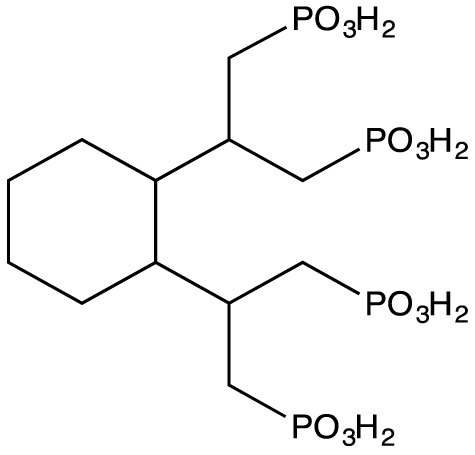
Chemical structure of CDTMP.

CDTMP (Figure 1) was synthesized by single step method previously reported by Panwar et al. (14). In brief, to a solution of trans-CDTA (0.5 g, 1.37 mmol) dissolved in 10 mL toluene, phosphorus acid (0.48 g, 6.85 mmol) was added. The mixture was then refluxed at 100°C while phosphorus trichloride (0.75 g, 0.755 mmol) in a volume of 300 mL was added drop wise to the refluxing mixture. After 3 h, the solvent was removed followed by addition of deionized water. The filtrate was concentrated under vacuum. The concentrated product was then precipitated by addition of ethanol to obtain the pure product. The formation of final product CDTMP was confirmed by Mass and NMR spectroscopy.

### Gallium Complexation Studies

To a solution of the CDTMP, 25 mg (0.05 mmol) in water was added Ga(NO_3_)_3_, 15 mg (0.06 mmol) at pH adjusted to 5.5 using sodium acetate. The reaction mixture was then heated at 90°C and stirred for 8 h. It was then allowed to cool and passed through the C-18 cartridge to remove unreacted species. The mixture was then filtered through a 0.22 μm syringe filter, freeze dried, to yield a white solid with a purity of >95% (15). Formation of the complex was confirmed by Mass spectroscopy.

### Radiochemical Synthesis of [^68^Ga]-CDTMP

The ^68^Ge/^68^Ga generator was eluted according to manufacturer protocol (Eckert and Ziegler) with 5 mL of 0.1 N hydrochloric acid (330–520 MBq). 2 mL of above eluate was used for labeling. A stock solution of CDTMP (1 mg/mL water) was prepared, and a 200 µL aliquot was used in labeling studies. Labeling was carried out in Na-HEPES buffer with pH adjusted between 3 and 3.5. The reaction mixture was heated for 10 min at 80°C, passed through C-18 cartridge and eluted with ethanol. Radiolabeling yield was determined by ITLC/SG 1 cm × 8 cm strips (methanol/ammonium acetate 1 M; 1:1) for each step of the experiments.

### Radiochemical Synthesis of [^188^Re]-CDTMP

CDTMP (3–5 mg) was dissolved in 0.5 mL of 0.5 M bicarbonate buffer, 200 µL of 0.1 M ascorbic acid having pH 9, 0.5 mL of 0.9% saline, and 0.2 mL (100 mg, 0.54 mM, 148 MBq) of ^188^ReO^−4^ solutions were mixed in a 10 mL vial. To the 0.02 mL of stannous chloride (100 mg/mL in 10% acetic acid), concentrated HCl was added. Under nitrogen, the pH of the reaction mixture was adjusted to 7 with 1 M NaOH. Radiochemical purity of [^188^Re] complexes was characterized by paper chromatography studies. It is known that using acetone as a solvent in paper chromatography; the ligand complexes remain at the bottom. Under identical conditions, free ^188^ReO^−4^ moved toward the solvent front.

### Preclinical Studies

#### HAp Binding Studies

To evaluate HAp binding, 20 mg of synthetic HAp was allowed to incubate in 1 mL of isotonic saline for 24 h. Labeled complex in a volume of 50 µL was then added, subjected to vortex for 10 s and incubated at ambient temperature for 10 min. After incubation, samples had been centrifuged, and the supernatant was removed. Hap fraction was washed twice with 0.5 mL saline, and all the washes were collected. Radioactivity was then checked in combined liquids and HAp fraction using gamma counter. Labeled complex binding of [^68^Ga]-CDTMP and [^188^Re] absorbed to HAp was determined (16).

#### Blood Clearance Studies

Blood clearance studies of [^68^Ga]-labeled complex were performed in normal New Zealand rabbit (*n* = 3) weighing 2.5–3.0 kg. 20 MBq of labeled complex [^68^Ga]-CDTMP was administered intravenously through the dorsal ear veins of rabbits, and blood samples (200 µL) were withdrawn at different intervals of time. The activity in blood, concerning percentage-administered dose, for different time intervals, was calculated by counting the samples in well counter and time activity curve was generated.

#### Serum Stability Studies

Serum stability of a radiolabeled pharmaceutical is related to various parameters such as pH, the presence of binding proteins, and metal metabolism. Serum was separated from blood by allowing the blood to clot for 1 h at 37°C in a humidified incubator. Then, the samples were centrifuged at 400 *g* and plasma separated into sterile tubes. To the 800 µL of serum, 200 µL of the [^68^Ga]-CDTMP complex was added and incubated at 37°C. Free gallium percentage was assessed by ITLC-SG using 1:1 ammonium acetate:methanol as mobile phase at different intervals of time.

#### Scintigraphy Studies

Scintigraphy studies of both the complexes [^68^Ga]-CDTMP and [^188^Re]-CDTMP were carried out in New Zealand rabbit by intravenous injection of 37 MBq of labeled complex through the ear vein. The acquisition was performed at 45 min post-intravenous injection for [^68^Ga] CDTMP and 1 h postinjection for [^188^Re]-CDTMP.

#### Biodistribution Studies

Biodistribution experiments of both [^68^Ga]-CDTMP and [^188^Re]-CDTMP were carried out in male BALB/c mice (*n* = 5 for each time point) weighing 25–30 g. 3.7 MBq of labeled [^68^Ga]-CDTMP complex was injected intravenously into the tail vein of each animal. The animals were dissected at particular time intervals up to 2 h postinjection, and desired organs were collected for [^68^Ga]-CDTMP and for [^188^Re]-CDTMP, the biodistribution was carried out up to 96 h at specific time intervals. The required organs were removed, made free from adhering tissue, and washed with normal saline. The radioactivity in each organ was counted using gamma counter. The data are expressed as the percent-administered dose per gram of the organ.

#### Myelotoxicity Studies

Myelotoxicity studies were performed on male Wistar rats. Myelotoxicity was evaluated by blood cell count (RBC, WBC, and platelets) post 75 MBq [^188^Re]-CDTMP intravenous injection at selected time points up to 16 days. Blood samples were withdrawn using heparinized capillaries in EDTA coated vials, and blood cell count was recorded on an automatic analyzer (CellTac α MEK6450K, Nihon Kohden).

#### Decay Correction

All the data including blood clearance studies, biodistribution, and imaging were subjected to decay correction.

## Results

### Radiolabeling

**Figure 2:**
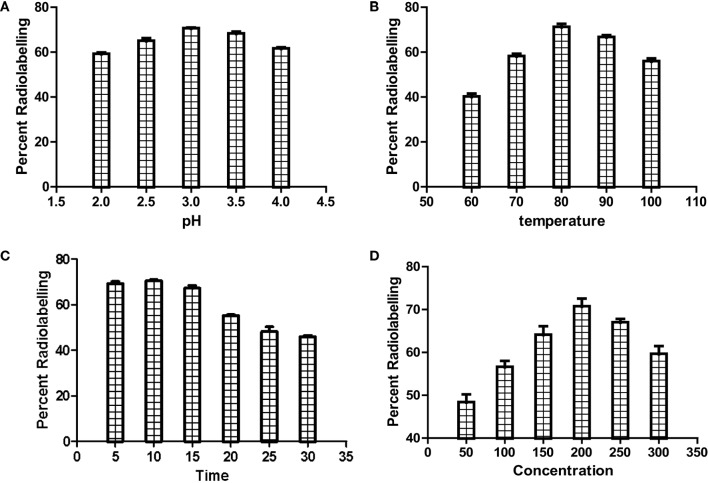
Optimization of radiolabeling of [^68^Ga]-CDTMP for **(A)** pH, **(B)** temperature, **(C)** heating time, and **(D)** compound concentration.

Before radiocomplexation of CDTMP, cold complexation of CDTMP was performed with cold gallium nitrate, which was characterized by mass spectroscopy (see Figure S1 in Supplementary Material). Radiochemical yield of [^68^Ga]-CDTMP was optimized for various conditions of pH, temperature, and compound concentration chromatographically using ITLC-SG (Figure 2). The optimum pH for labeling was 3–3.5, and deterioration of radiolabeling yield was observed at higher pH. The radiolabeling efficiency was found to be 70 ± 1%. [^188^Re-CDTMP] too was labeled with radiochemical purity higher than 97 ± 1.2%. It was found that addition of 100 µL of 0.1 M ascorbic acid increases the stability of complex. Radiochemical purity for [^68^Ga]-CDTMP was assessed using EZ-TLC Scanner (see Figure S2 in Supplementary Material) Radio-HPLC analysis after tc-18 cartridge purification was performed for [^68^Ga]-CDTMP, which showed the presence of only one radiolabeled species at a retention time of 4.35 min (see Figure S3 in Supplementary Material). UV Absorption spectrum was acquired for both the compounds. No absorption was observed for both CDTMP and col gallium CDTMP complex [Ga(III)-CDTMP] (see Figures S4A,B in Supplementary Material) due to the highly acidic nature of compound. Only radiochromatogram could confirm the formation of single species.

### HAp Binding and Serum Stability

**Figure 3:**
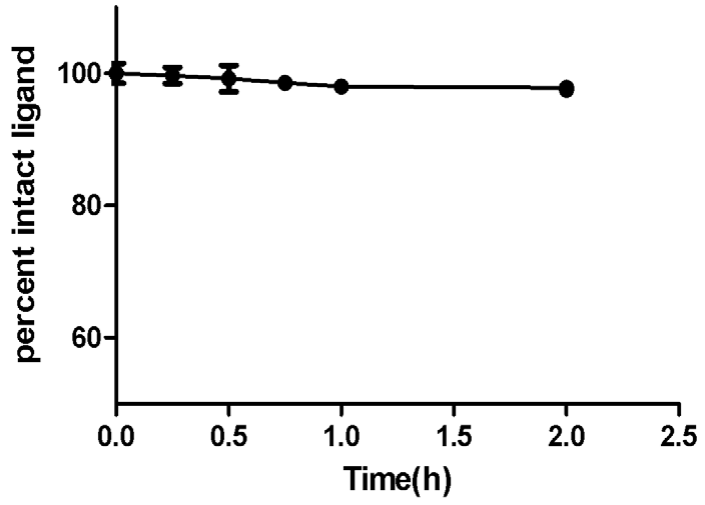
Percentage intact ligand as a function of time showing human serum stability of [^68^Ga]-CDTMP at physiological conditions.

*In vitro* binding study on synthetic HAp simulates the binding of CDTMP complex to osseous tissue. The [^68^Ga]-CDTMP binding to HAp was found to be 89 ± 3% whereas for [^188^Re]-CDTMP Hap binding observed was 82.7 ± 2%, which demonstrates an excellent absorption of labeled complexes to HAp. Binding studies showed that the labeled complex could be further applicable for further clinical studies. The labeled complex [^68^Ga]-CDTMP was found to be stable in serum as 97.5% remaining intact after 3 h (Figure 3).

#### Blood Clearance

**Figure 4:**
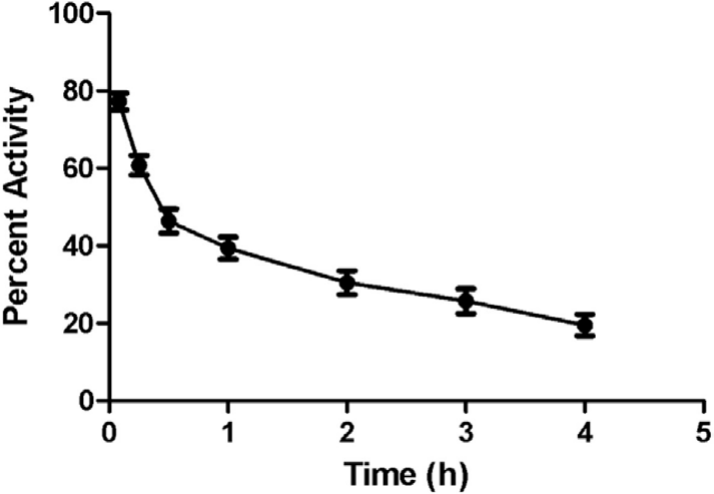
Time activity curve showing blood clearance of [^68^Ga]-CDTMP (20 MBq) postinjection in New Zealand rabbit.

Blood kinetic studies in rabbits showed that the complex [^68^Ga]-CDTMP followed a biphasic pattern of clearance. There was a rapid clearance of the [^68^Ga]-CDTMP from the body. After 1 h, the clearance followed a slow pattern, and at 4 h approximately 18% of the activity remained in the blood (Figure 4). The biological half-life was obtained to be *t*_1/2_ (fast): 12 min; *t*_1/2_ (slow): 3 h and 10 min. A rapid washout of [^68^Ga]-CDTMP favors the early imaging after administration as well as reduces the radiation burden to the subject.

#### Scintigraphy Studies

**Figure 5:**
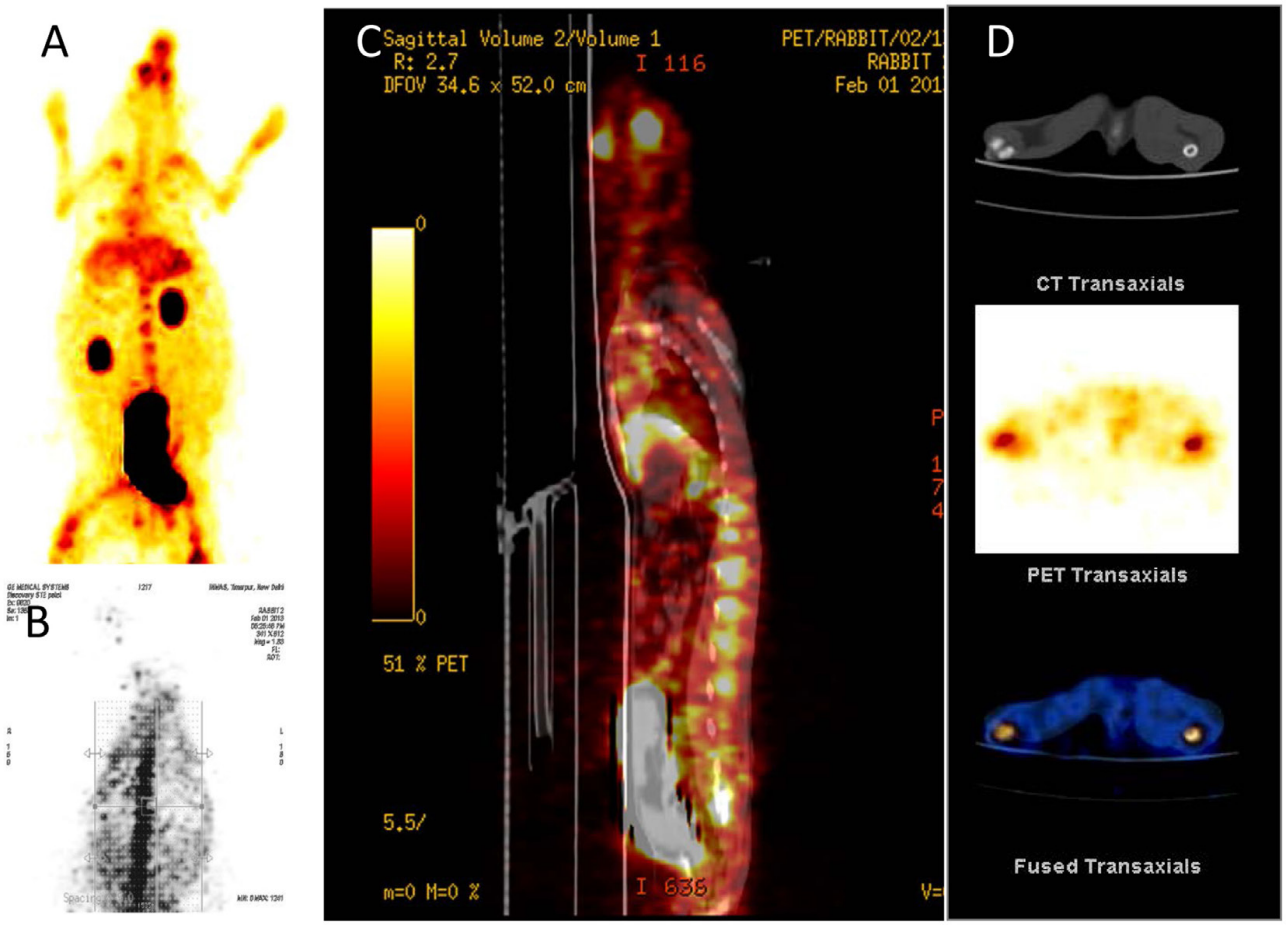
**(A)** [^68^Ga]-CDTMP anterior view 45 min p.i. **(B)** [^68^Ga]-CDTMP posterior view of positron emission tomography (PET) image in normal rabbit 45 min p.i. **(C)** Sagittal section of PET image. **(D)** CT transaxial PET transaxial and PET/CT-fused transaxial image confirming bone uptake.

**Figure 6:**
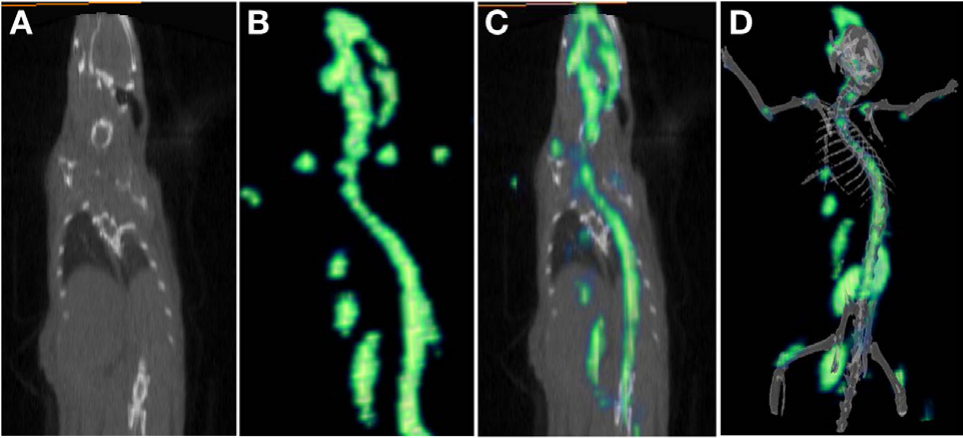
**(A)** CT frontal view. **(B)** [^68^Ga]-CDTMP frontal view of MicroPET image in Balb/C mice 45 min postinjection. **(C)** PET/CT-fused frontal view image. **(D)** 3D MicroPET/CT (volume rendered CT image) coregistered image confirming bone uptake.

**Figure 7:**
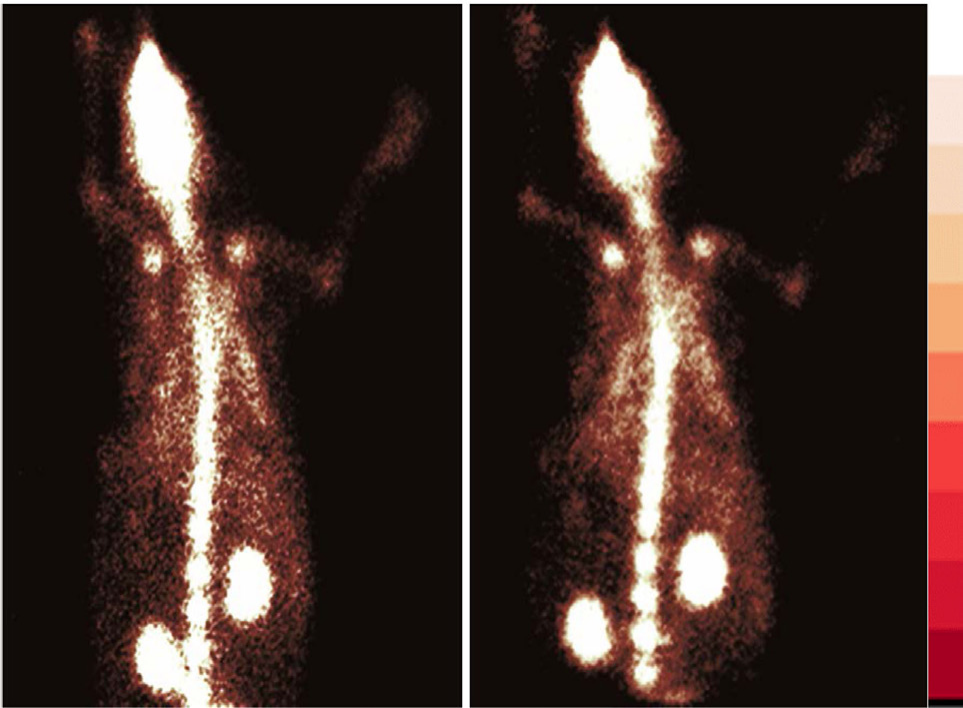
Whole body γ scintigraphy of [^188^Re]-CDTMP in rabbits l h postinjection (*n* = 2).

[^68^Ga]-CDTMP PET and MicroPET imaging were performed on New Zealand rabbit and Balb/C mice, respectively. Imaging was done at 45 min postinjection for both mice and rabbit. High bone uptake as compared to contralateral tissue was observed, which attributes to its use as a bone imaging agent. Both PET and MicroPET images were validated by CT, and fused PET/CT images were also obtained (Figures 5 and 6). Volume of interest analysis of the PET showed a bone to muscle ratio of 21.258 ± 1.3. Semiquantitative analysis of the MicroPET images depicts bone to muscle ratio of 24.146 ± 0.98, bone to kidney ratio of 7.976 ± 0.78. Imaging the [^68^Ga]-CDTMP uptake in a healthy animals both mice and rabbits showed that the tracer accumulation in bones was prominent. Under physiological conditions, a high activity in the joints was observed. Thus, the [^68^Ga]-CDTMP accumulation observed was greater in the shoulder and along the backbone as shown in [^68^Ga] PET and MicroPET images suggestive of preferential accumulation of radiotracer in the metabolically active regions of bone. Scintigraphic images of [^188^Re]-CDTMP for skeletal uptake in rabbits were taken at 1, 2, and 24 h postinjection. Images revealed high selective skeletal uptake with insignificant non-osseous tissue accumulation (Figure 7).

#### Biodistribution Studies

**Figure 8:**
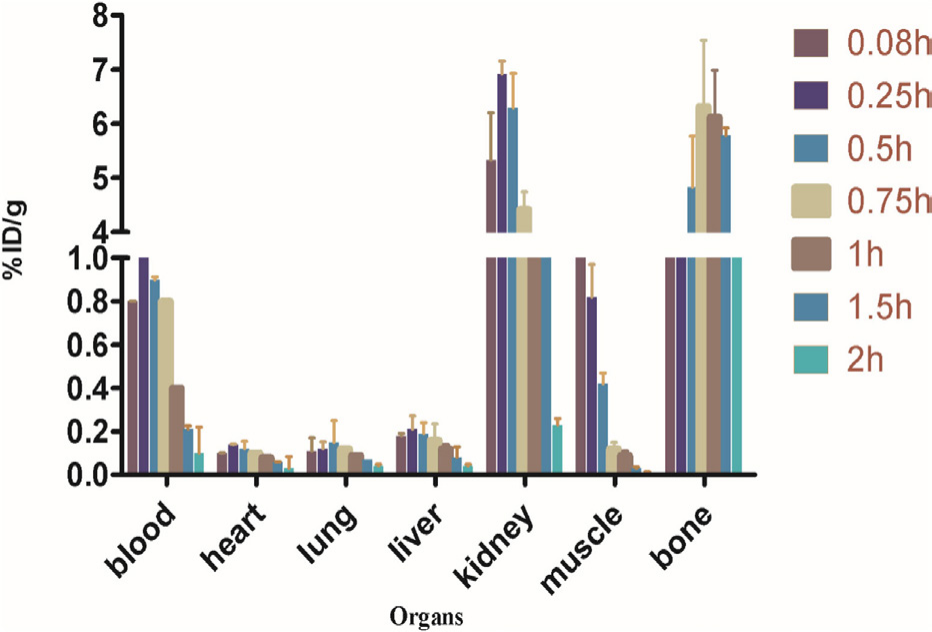
Biodistribution of [^68^Ga]-CDTMP in Balb/c mice. Data expressed in %ID/g ± SD from five animals.

**Figure 9:**
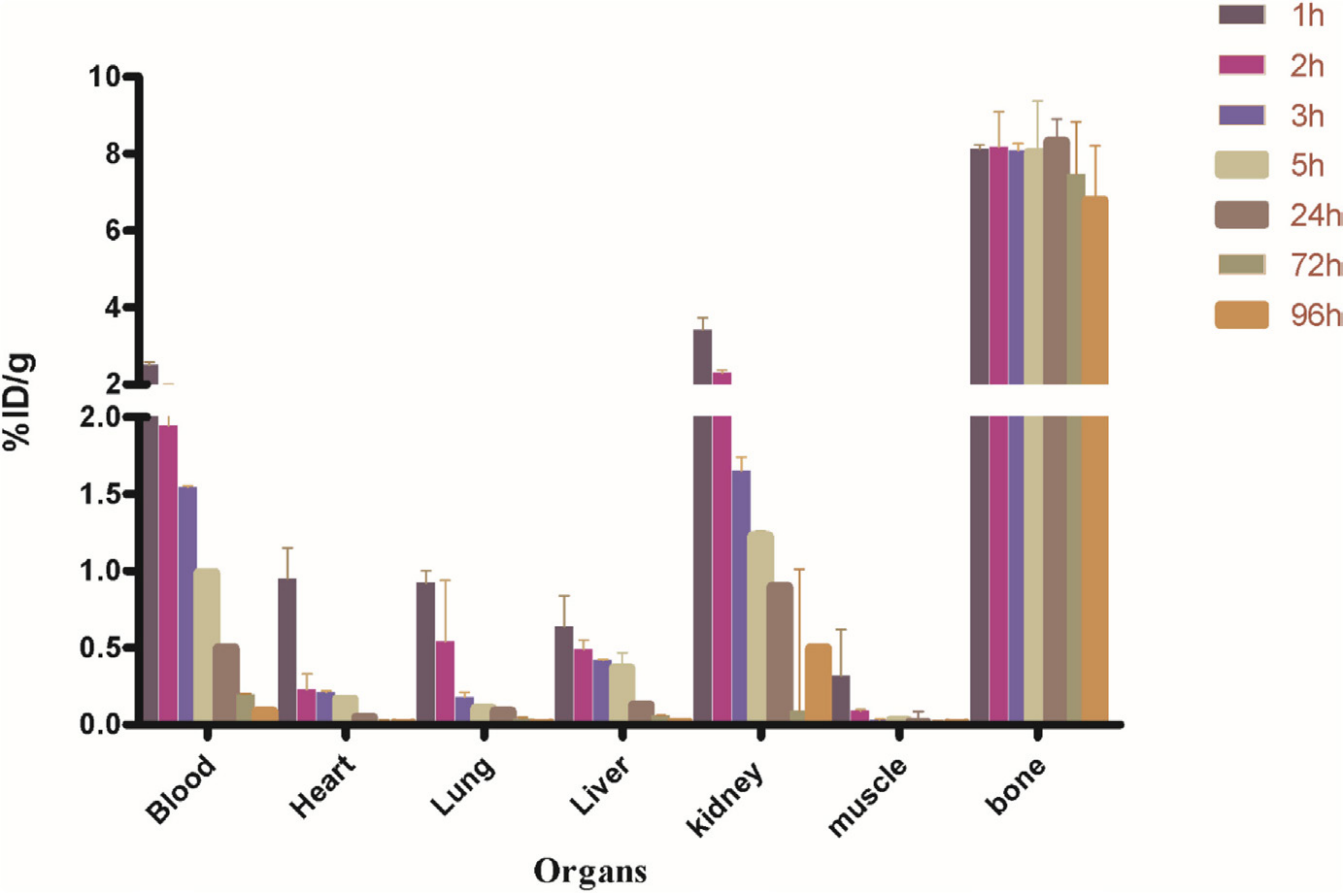
Biodistribution of [^188^Re]-CDTMP in Balb/c mice. Data expressed in %ID/g ± SD from five animals.

**Figure 10:**
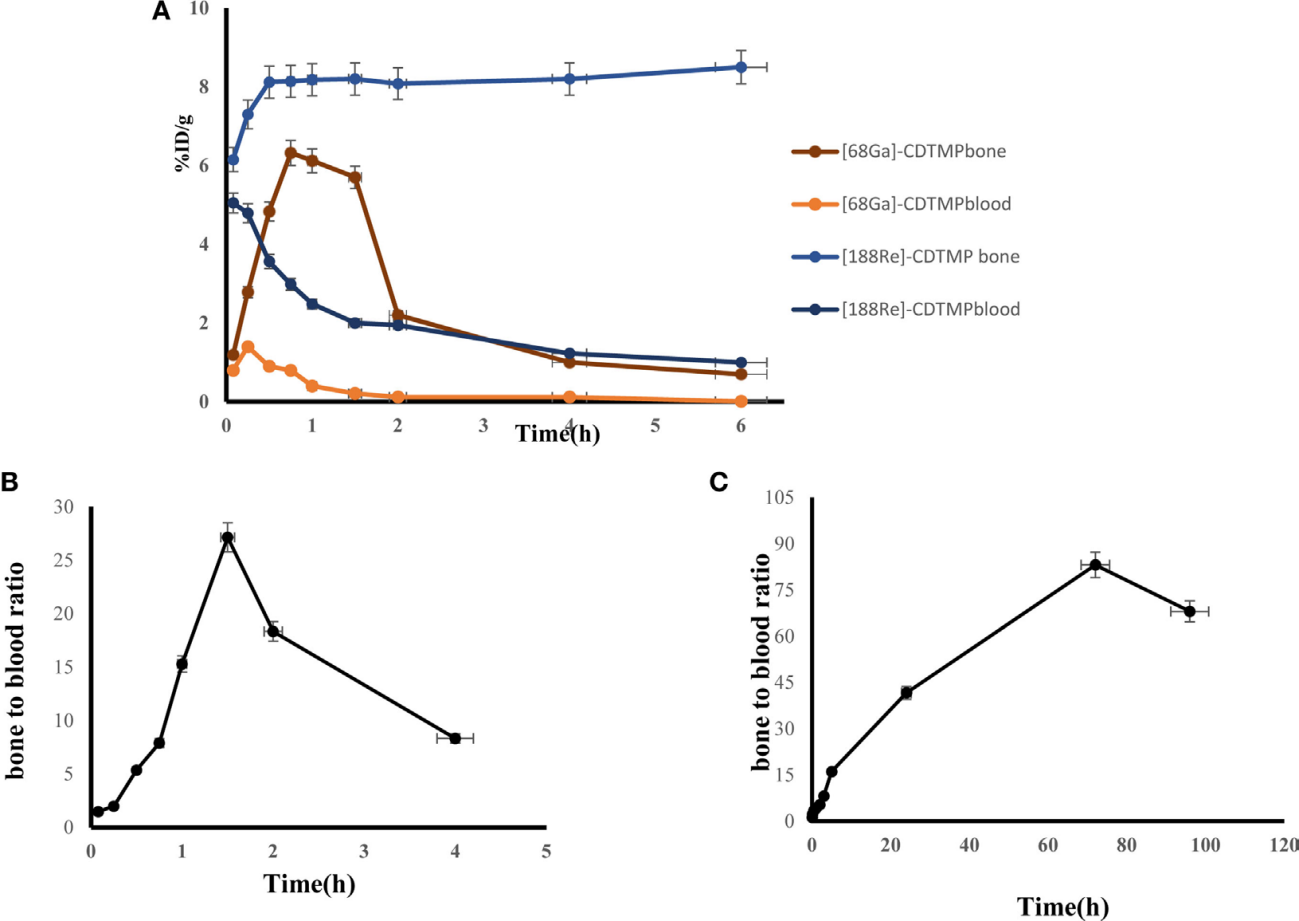
**(A)** Line graph depicting dynamic behavior of [^68^Ga]-CDTMP and [^188^Re]-CDTMP in blood and bone tissue in mice. **(B)** Bone to blood ratio as a function of time for [^68^Ga]-CDTMP. **(C)** Bone to blood ratio as a function of time for [^188^Re]-CDTMP.

Biodistribution experiments for [^68^Ga]-CDTMP carried out in mice showed maximum bone uptake of 6.12 ± 1.22 at 45 min postinjection (Figure 8). High kidney uptake observed for both the complexes reveals the renal route of excretion. For [^188^Re]-CDTMP, total skeletal uptake was 8.12 ± 1.11%ID/g observed at 1 h from biodistribution studies (Figure 9). Biodistribution data for blood for both radiolabeled complexes depict a shorter biological half-life and faster clearance for [^68^Ga]-CDTMP and a longer biological half-life for [^188^Re]-CDTMP (Figure 10A). Bone to blood ratio was also calculated for both the radiolabeled complexes, which clearly showed significant bone uptake (Figures 10B,C).

#### Myelotoxicity Studies

In myelotoxicity studies after 4 h, 1, 3, and 5 days of [^188^Re]-CDTMP injection in Wistar rats, there was a slight decrease in white blood cell counts of the animals postinjection (Table 1).

**Table 1:** Myelotoxicity of [^188^Re]-CDTMP.

	RBC (×10^9^/dL)	Hb (g/L)	WBC (×10^9^/dL)	Platelet (×10^9^/dL)
C	5.8 ± 0.98	147 ± 18.0	11.8 ± 3.4	330.01 ± 10.5
4 h	4.40 ± 1.2	132 ± 0.18	8.1 ± 1.2	220.6 ± 12.0
1 day	4.4 ± 1.6	139 ± 0.18	8.58 ± 0.9	228.3 ± 15.2
3 days	4.69 ± 1.32	138 ± 0.18	8.8 ± 1.5	236.01 ± 17.5
5 days	4.88 ± 0.9	142 ± 0.18	8.8 ± 1.9	237.01 ± 14.1
7 days	5.33 ± 0.98	142 ± 0.18	9.1 ± 1.1	260.1 ± 24
16 days	5.4 ± 1.1	144 ± 0.18	9.7 ± 2.1	278.01 ± 11

After 7 days, there was no significant difference between RBC and Hb from baseline. In the hematological evaluation, platelets were found to be the most sensitive cells. With every therapeutic dose of [^188^Re]-CDTMP, a decrease in the number of platelets was observed. Insignificant decrease of platelet counts was noted in the first period (7 days), which was found to recover in a period of 16 days. All variation values obtained were within the normal range. No significant deviation was detected in the red blood cells parameter. The drop in circulating WBC and platelets occurs from 4 h to 7 days. After 16 days, both the white blood cell and platelet counts increased with time. No evidence of destruction and death of vital organs were observed.

## Discussion

Development of [^68^Ga]-labeled bone-seeking agents could lead to further improvement in bone imaging using PET independent of cyclotron-based radiopharmacy. After an easy and short radiolabeling procedure, [^68^Ga]-CDTMP forms a stable complex assessed *in vivo* and thus has a good potential for use as radiotracer. An easy, fast and reliable kit-based preparation protocol could enable PET facilities without on-site cyclotron to perform PET bone scans. In clinical scenario, [^99m^Tc]-MDP and [^99m^Tc]-HMDP are widely accepted bone scintigraphy agents, but their optimized chemical and pharmaceutical perspective have not yet been optimized as these complexes are not pure single chemical species but are mixtures of short-chain and long-chain oligomers (17). Synthetic and radiosynthetic ([^68^Ga]-CDTMP and [^188^Re]-CDTMP) approach reported that CDTMP eliminates the formation of undesired intermediate products thereby obviating the need for its further purification.

One of the major factor considered for selective localization of a radiotracer in bone is active bone mineralization of HAp [Ca_10_(PO_4_)_6_(OH)_2_], which is the main mineral constituent of bones. HAp-binding assay was carried out to evaluate phosphonates interaction with skeletal tissue. The uncontrolled change in mineralization of HAp is the basis for phosphonates bone imaging. HAp-binding studies were performed to assess the adsorption behavior of radiolabeled complex. Gallium-based radiopharmacy of CDTMP overcomes the need of in-house cyclotron facility as well as enhanced spatial resolution, and quantification properties of PET technique are much superior to conventional SPECT technique. Much advanced imaging results of PET/CT, as compared to SPECT/CT, are clearly demonstrated by using [^18^F]-fluoride (18).

^68^Ga possess a half-life of 68 min; positron emission fraction of β^+^89%; and E^+^ β max 1.9 MeV is a positron-emitting nuclide reported with a remarkable clinical imaging potential, as the radionuclide can be easily obtained on-site from ^68^Ge/^68^Ga generators (19–21). A review of published reports of bone-seeking agents such as [68Ga]-labeled citrate (22), [68Ga]-labeled tripolyphosphate (23), [^18^F] and [^68^Ga]-labeled ethylenediamine tetramethylene phosphonate (24–27) bone-seeking agents did not validate sufficient potential for their clinical application.

Rhenium nuclides have γ emissions, which allow for easy detection of distribution pattern and clearance by gamma scintigraphy. The success of the bone pain palliation therapeutic treatment by a particular radiopharmaceutical depends upon the activity administered and percentage uptake of bone. The radioactivity administered and the extent of radiopharmaceutical uptake by bone are the two most important properties considered as the determinants of the success of the treatment with therapeutic radioisotope. To evaluate the skeletal uptake of the radiolabeled phosphonates used in this study, we employed the method that is based on conventional ROI technique and allows compartmental calculation for bone and soft tissue. The preclinical evaluation shows its therapeutic efficacy as [^188^Re]-CDTMP. [^188^Re-CDTMP] was labeled with radiochemical purity higher than 97% and showed a bone uptake of ~70%. The evaluated compound CDTMP showed fast and high bone absorption and moderate soft tissue accumulation in soft tissue. The renal clearance time proved to be longer for [^188^Re-CDTMP], and a high activity was also observed in the skeleton was constant for 96 h, which clearly reveals the long biological half-life of [^188^Re-CDTMP] in the target tissue. Similar results were also observed for [^177^Lu]-DOTAZOL and [^68^Ga]-DOTAZOL (28). The rapid accumulation of agent in bone and high target-to-non-target uptake ratio showed that CDTMP has both therapeutic potential as when coupled to β-emitting radionuclides.

The present study suggests that [^68^Ga]-labeled CDTMP may serve as a promising vector in nuclear medicine for PET imaging and bone pain palliation with a good bone accumulation property. *In vivo* PET evaluation revealed predominant uptake in bone with low non-target accumulation and rapid non-osseous clearance. Apart from present assessment, future clinical prospects of [^68^Ga]-CDTMP require further evaluations and preclinical trials. Moreover, the present study also describes the therapeutic potential of [^188^Re]-CDTMP as a good therapeutic probe in preclinical studies. Being generator-based radioisotopes ^68^Ga and ^188^Re have easy availability; single step synthetic strategy of CDTMP both facilitates an on-site simple radiopharmacy with low costs.

## Ethics Statement

This study was carried out in accordance with the recommendations of (CPCSEA Regn No.8/GO/RBi/S/99). The protocol was approved by the INMAS Institutional Animal Ethics committee (CPCSEA Regn No.8/GO/RBi/S/99).

## Author Contributions

PH participated in research design, conducted experiments, performed image processing and data analysis, and contributed to the writing of the manuscript. AM participated in research design, contributed new reagents, and contributed to the writing of the manuscript. AmJ conducted experiments and contributed to the writing of the manuscript. SP, VM, and AnP conducted experiments. BS participated in research design and contributed to the writing of the manuscript.

## Conflict of Interest Statement

The authors report no conflict of interest. The authors alone are responsible for the content and writing of the paper.
