# Supplementary material for: [68Ga]/[188Re] Complexed [CDTMP] Trans-1,2-Cyclohexyldinitrilotetraphosphonic Acid As a Theranostic Agent for Skeletal Metastases
Source: Front Med (Lausanne). 2017 Jun 9;4:72. doi: 10.3389/fmed.2017.00072 (PMC5465288; doi:10.3389/fmed.2017.00072)

FigureS1: Mass Spectra of Ga(III)-CDTMP

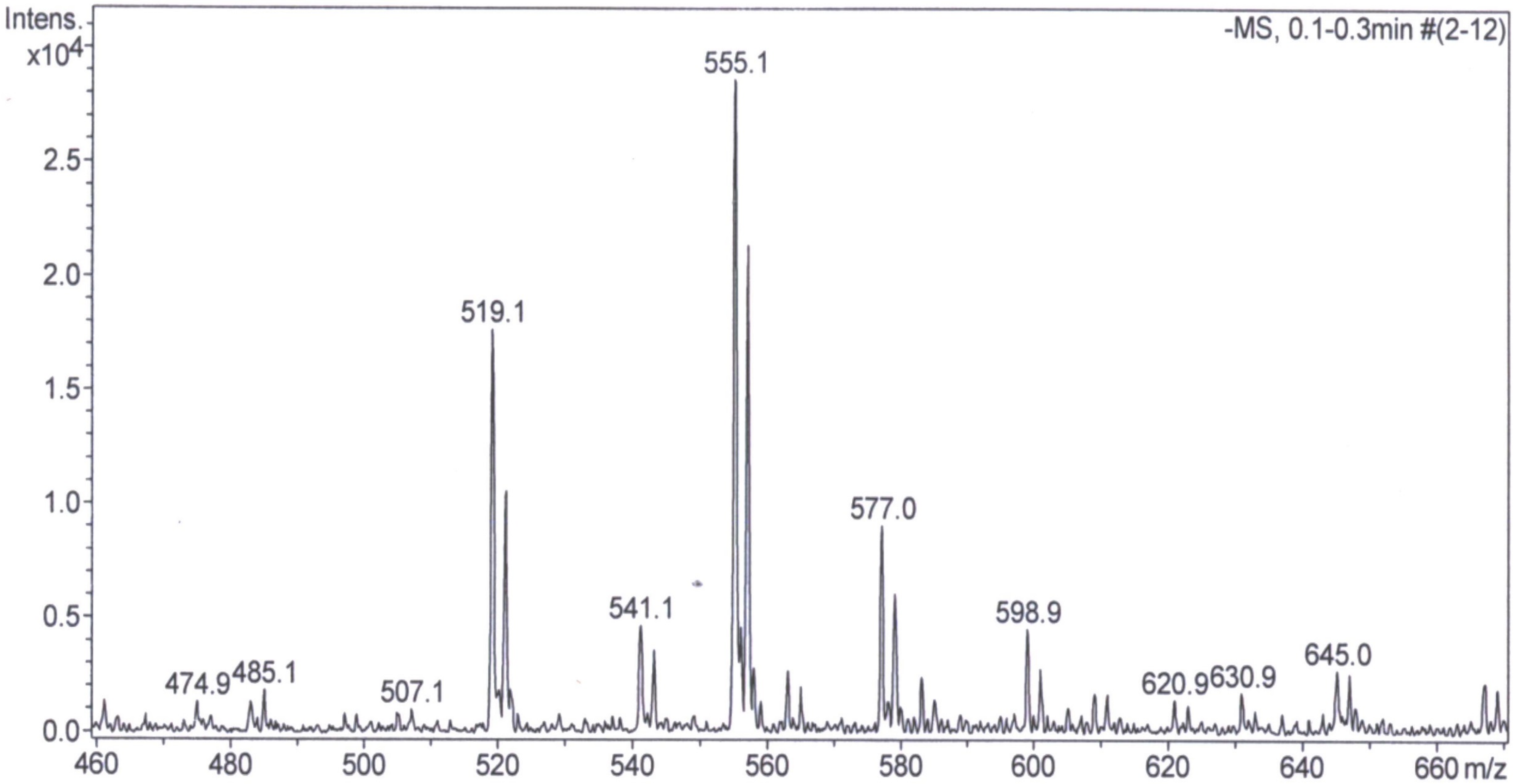

FigureS2: EZ-TLC profile s of [^{68}Ga]-CDTMP , and mixture of[^{68}Ga]-CDTMP and Free Ga-68 in 1:1 Ammonia:Methanol solvent system

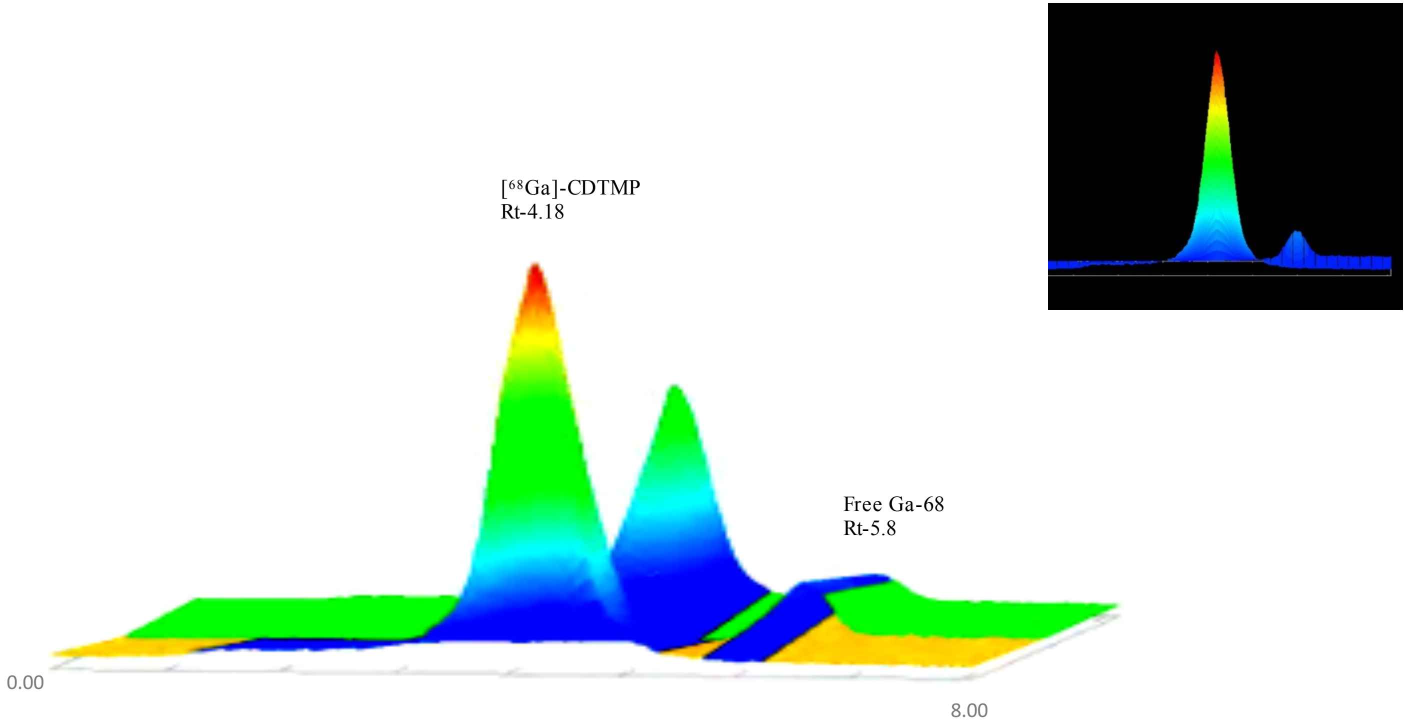

FigureS3: radio-HPLC chromatogram confirming formation of [68Ga]-CDTMP

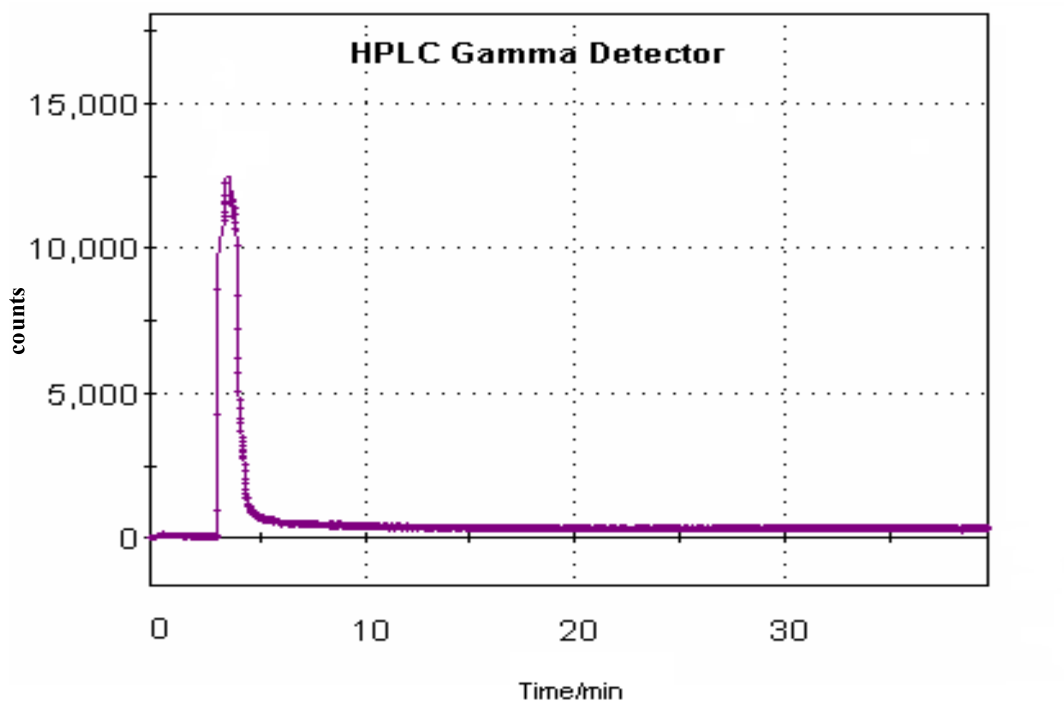

FigS4a: UV Absorption Spectra of CDTMP

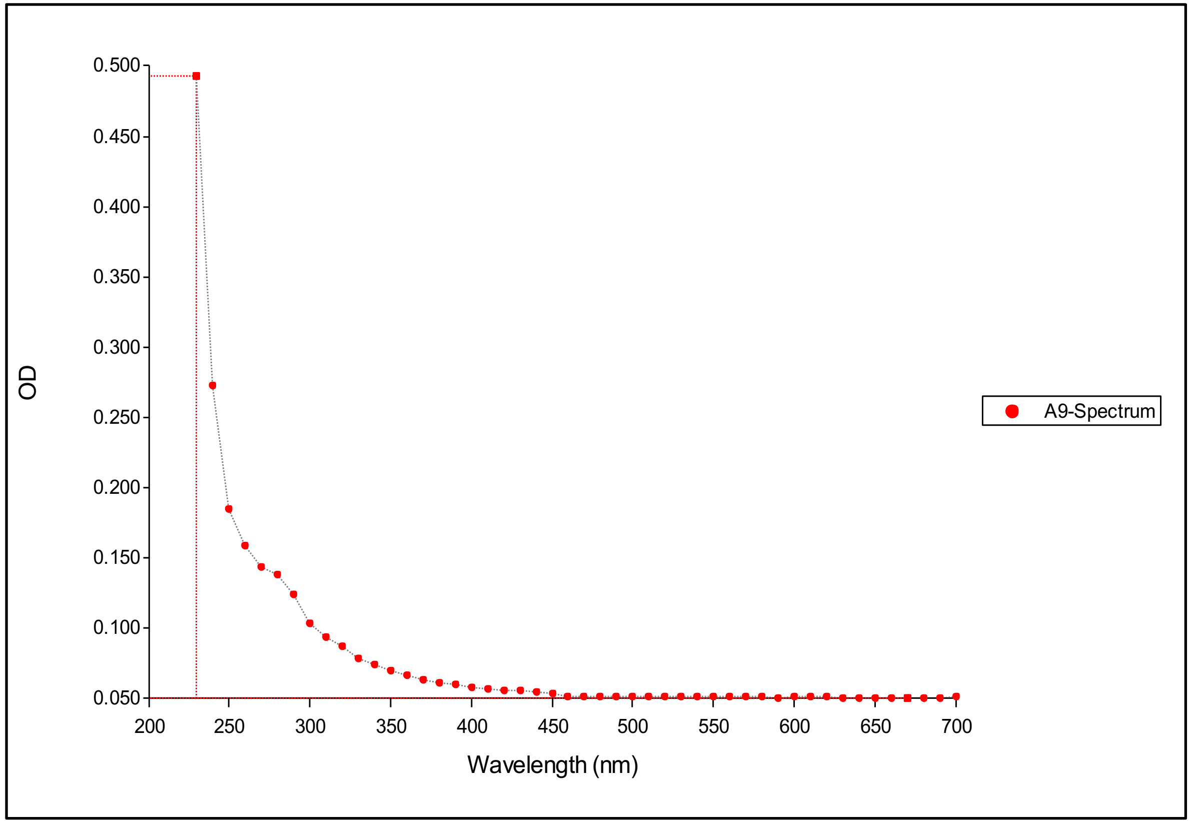

FigureS4b:UV Absorption Spectra of Ga(III)-CDTMP

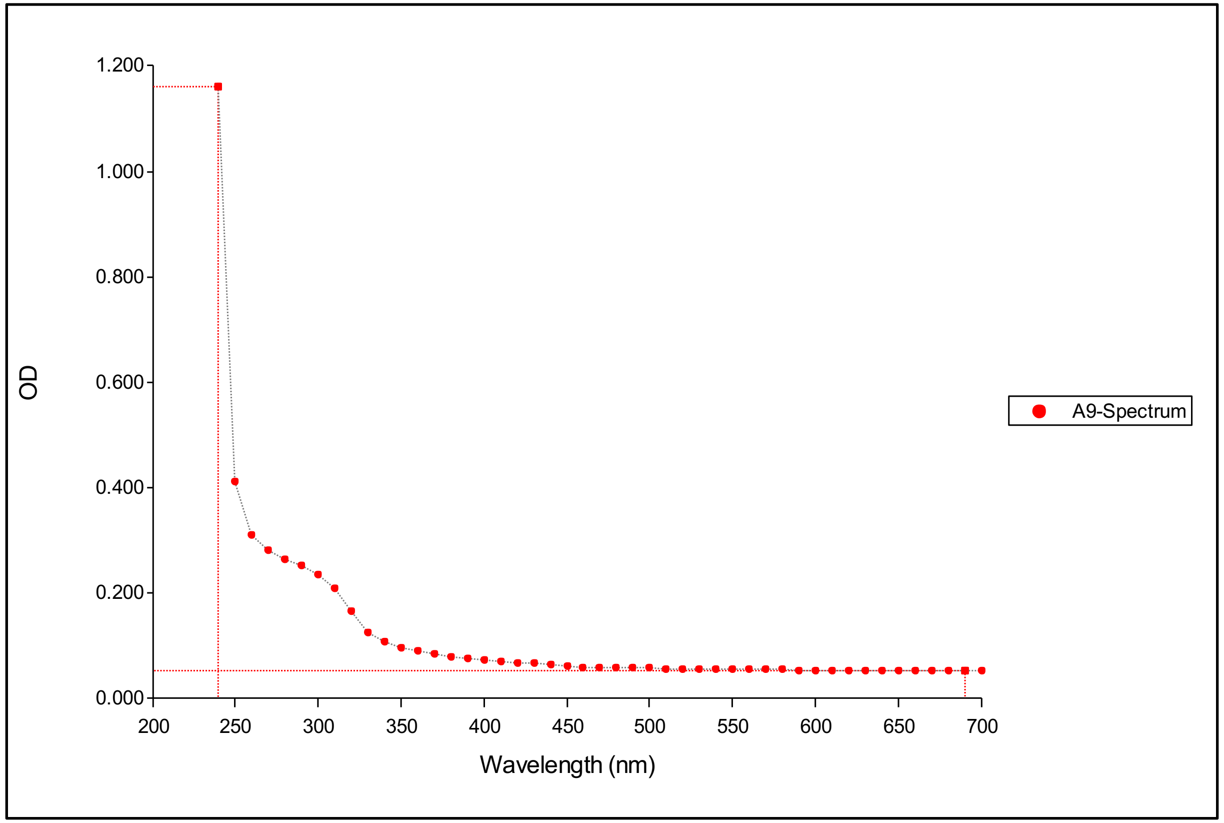

Supplement: Supplementary file 1 [file Presentation_1.PDF]
